# Fabrication of multifunctional titanium surfaces by producing hierarchical surface patterns using laser based ablation methods

**DOI:** 10.1038/s41598-019-43055-3

**Published:** 2019-04-30

**Authors:** Christoph Zwahr, Ralf Helbig, Carsten Werner, Andrés Fabián Lasagni

**Affiliations:** 1 Institute of Manufacturing Technology, Technische Universität Dresden, George-Bähr Str. 3c, 01069 Dresden, Germany; 2 Fraunhofer-Institut für Werkstoff- und Strahltechnik (IWS), Winterbergstraße 28, 01277 Dresden, Germany; 3 Institute of Biofunctional Polymer Materials, Leibniz-Institut für Polymerforschung Dresden e. V., Hohe Straße 6, 01069 Dresden, Germany

**Keywords:** Biomaterials - cells, Ultrafast lasers

## Abstract

Textured implant surfaces with micrometer and sub-micrometer features can improve contact properties like cell adhesion and bacteria repellency. A critical point of these surfaces is their mechanical stability during implantation. Therefore, strategies capable to provide both biocompatibility for an improved implant healing and resistance to wear for protecting the functional surface are required. In this work, laser-based fabrication methods have been used to produce hierarchical patterns on titanium surfaces. Using Direct Laser Writing with a nanosecond pulsed laser, crater-like structures with a separation distance of 50 µm are produced on unpolished titanium surfaces. Directly on this texture, a hole-like pattern with 5 µm spatial period is generated using Direct Laser Interference Patterning with picosecond pulses. While the smaller features should reduce the bacterial adhesion, the larger geometry was designed to protect the smaller features from wear. On the multifunctional surface, the adherence of *E. Coli* bacteria is reduced by 30% compared to the untreated reference. In addition, wear test performed on the multiple-scale patterns demonstrated the possibility to protect the smaller features by the larger craters. Also, the influence of the laser treatment on the growth of a titanium oxide layer was evaluated using Energy Dispersive X-Ray Spectroscopy analysis.

## Introduction

According to actual market research, titanium and its alloys are still the most used materials in dental implant industry^1^. This is due to their good tolerance by living tissues as well as their capability of osseointegration promotion^2,3^. Nevertheless, the satisfactory integration of the implant with the surrounding bone tissue is determined by the physical and chemical properties of its surface^4,5^. Despite a lot of different processes to modify metallic implant surfaces, still approximately 30% of patients develop a disease known as peri-implantitis. This disease is an oral inflammation, characterized by loss of supporting bone and even the possible loss of the implant^6^. This can be caused by inappropriate surgical technique or poor bone-implant interaction^7,8^. The latter can be counteracted by roughening the implant surface which can improve the healing of the bone^9,10^. This has been for instance confirmed in a rabbit animal model, were an 18-fold increase in tear strength between the implant and the bone was achieved by increasing the average roughness from 10 μm to 50 μm^11^.

In addition to the surface roughness, also relevant for a good biocompatibility of the titanium implant with the leaving tissue is the presence of a thick enough titanium oxide layer (TiO_2_). This layer can pacify tissue-destroying agents immediately after implantation^3^. Furthermore, It has been observed, that in humans, the thickness of the Ti oxide layer on dental implants increases from 5 nm to 200 nm in 6 years of osseointegration^12,13^. This is of high importance since the porous oxide layer allows the incorporation of bone calcium and phosphorus ions within this layer which continues naturally during the implant lifetime in the body. In contrast, the oxide layers on faulty implants were of same thickness or thinner than usual during the first days of osseointegration. Due to this, deosseointegration is associated with the loss of the biocompatible interface, formed by the Ti oxide surface layer^3,14,15^.

Decisive for the performance of a titanium implant is also its ability to prevent bacterial adhesion (antibacterial properties), especially close to the gingiva zone, because bacteria can reach the implant and later the bone interface^3,16^. Thus, the implant abutments are usually high gloss polished with surface roughness in the sub-µm level (S_a_ < 0.2 µm)^17,18^. However, a more promising strategy to achieve an antibacterial property is to produce micrometer and sub-micrometer features on the implant surface, as it has been recently demonstrated^19–23^. In this frame, it has been observed that structures with features that are slightly smaller than the bacteria size provided the best results in terms of bacteria repellency due to the reduction of the cell-surface contact. In addition, hole/crater-like structures that are slightly larger than the bacteria size can inhibit the possibility of forming colonies and also exhibit antibacterial properties.

Promising methods for forming antibacterial surface patterns are laser processing techniques such as ultrashort pulsed laser irradiation, laser interference lithography (LIL) and direct laser interference patterning (DLIP)^20–26^ In the case of ultrashort pulsed laser irradiation, a single laser beam, with fs or ps laser pulses, is passed over the materials surface, creating for instance Laser-Induced Periodic Surface Structures (LIPSS). These self-organized structures can have feature sizes smaller than the laser wavelength used. Using this method, LIPSS with feature sizes between 0.5 µm and 0.9 µm permitted to reduce the bacteria adhesion of *E*. *Coli* on stainless steel by 99.8% compared to an untreated reference with 0.37 µm average surface roughness^23^.

In case of DLIP, interference patterns produced by overlapping two or more laser beams on the sample permit to create a periodic pattern at the surface of the irradiated material^27–29^. This method showed the possibility to reduce the bacterial adhesion of *Staphylococcus Epidermidis* and *Escherichia Coli* by 50% and 60%, respectively, when producing a hole -like structure of 0.5 µm spatial period on SU-8 photoresist. In contrast, it was also shown that the highest retention or adhesion of bacteria was achieved for periodic surfaces with repetitive distances (spatial period) of 1 µm, that was in the range of the bacteria size, independently of the geometry of the periodic structure (line-like, hole-like or pillar-like). However, not only patterns with features sizes smaller than the bacteria size have shown to be capable of reducing the adhesion of bacteria. For instance, hole-like patterns with a 5 µm spatial period permitted to decrease the bacteria adhesion also by about 50% for *S*. *Epidermidis* as well as for *E*. *Coli* compared to a flat reference^22^.

However, in addition to antibacterial properties, micro-patterns on metallic implants must have a certain stability to mechanical damage because they need to survive the implantation. The stability of laser-machined micro-patterns on steel and titanium has been studied in the past, with the focus on wear and friction reduction^30,31^. Although friction could be reduced by using laser patterning, it was also shown that the produced micro-patterns with feature sizes between 9 µm and 0.3 µm, were easily damage and thus not providing sufficient wear resistance.

An approach to protect small antibacterial patterns has been described by Roessler *et al*. on polyimide substrates^32^. By using DLIP, two-level hierarchical micro-patterns were fabricated to protect the smaller features in the cavities of the larger ones. They also showed that crater-like structures have a better mechanical stability than pillar-like like ones.

As it can be seen from the above described state of the art, there is still a necessity to produce multifunctional surface patterns capable to provide both, bacteria repellence as well as mechanical stability to mechanical damaging, which determined the main objective of this here presented work. In consequence, in this study, rough blanks produced from pure titanium (grade 4) are hierarchical patterned by combining DLW and four-beam DLIP in order to produce multifunctional surfaces. Pure titanium has excellent biocompatibility and, unlike for some Ti alloys, no toxicity has been reported^33^. Compared to other pure titanium materials, grade 4 has the highest mechanical strength (~550 MPa)^33,34^. The samples are firstly structured using a ns pulsed laser source with a laser scanning system to obtain crater-like structures separated by 50 µm. After that, they are treated with dot-like interference pattern with a pulsed ps laser source. The resulting topography, morphology and chemistry of the treated Ti surfaces are analyzed by confocal microscopy, scanning electron microscopy (SEM) and energy dispersive X-ray spectroscopy (EDX). The potential for improving antibacterial properties with this technology is evaluated by bacterial adhesion tests of *E*. *coli*. The resistance of the surface pattern against mechanical damage is measured by wear tests.

## Results and Discussion

### Fabrication of crater-like structures using DLW

**Figure 1:**
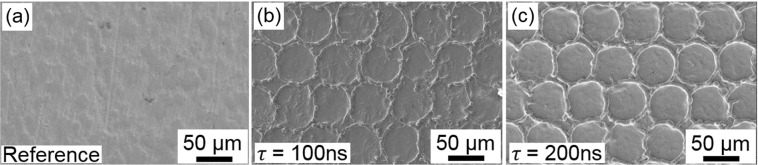
Scanning Electron Microscope images of titanium surfaces. (**a**) Untreated Ti surface (**b**) DLW processed surface with 100 ns pulses. (**c**) DLW processed Ti surface with 200 ns pulses. The patterns have a spatial period of 50 µm and were fabricated using laser fluences of (**b**) 3.0 J/cm^2^ and (**c**) 5.9 J/cm^2^, respectively.

In a first set of experiment, the Ti samples (Fig. 1a) were treated using Direct Laser Writing (DLW) to produce the large scaled microstructures. Figure 1b,c show exemplary Ti blanks that have been patterned to produce crater-like geometries with 50 μm spatial period using DLW. The targeted DLW structure in this experiment fulfills different tasks. Firstly, it should improve the adhesion with the bone-cells as already demonstrated for instance by Hallgren *et al*.^35^. Secondly, they should also protect the smaller features (to be described in the next section) as well as the thin Ti oxide layer from mechanical damage^35^.

The structure morphology was varied using different pulse durations between 100 ns and 200 ns, since it is well known that the pulse duration can strongly affect the heat affected zones as well as the amount of material molten during the laser treatment (see Fig. 1b,c, for 100 and 200 ns pulses, respectively). Each crater was produced using a single shot. The reference surface that is shown in Fig. 1a, had an average surface roughness Sa of 0.4 µm, a root mean square height Sq of 0.5 µm and a peak-to-valley height Sz of approximately 5.2 μm.

By varying the laser fluence (energy density) the deepest craters were obtained at 3.0 J/cm^2^ and 5.9 J/cm^2^ for the 100 ns and 200 ns pulses, respectively. These laser fluence values are about ~1.8 times higher than the ablation threshold which are 1.6 J/cm^2^ and 3.5 J/cm^2^ for 100 ns and 200 ns pulses, respectively. The last where determined experimentally by plotting the squared diameter of the ablated areas as function of the pulse energy and extrapolating the diameters to zero^36^. In addition, the DLW laser treatment permitted to reduce the peak-to-valley roughness of the Ti material into the craters down to 4.1 ± 1.4 µm and 2.6 ± 0.1 µm for 100 ns and 200 ns pulse durations, respectively. The average surface roughness and the mean square height of the samples remained constant, with Sa = 0.4 ± 0.1 µm and Sq = 0.5 ± 0.1 µm for both pulse durations.

The reduction of the peak-to-valley roughness can be related to the melting of the initial surface roughness into the craters followed by the resolidfication of the material creating a smooth melt layer.

As it well know, the diffusion of the heat into the material can be calculated using the thermal diffusion length *l*_*T*_ according to Eq. 1:

1 $${l}_{T}=\sqrt{\frac{K\cdot \tau }{\rho \cdot {c}_{p}}}$$

where *τ* is the pulse duration of the laser source, *K* is the thermal conductivity, *ρ* is the density and *c*_*p*_ the specific heat capacity of the material.

As it can be observed, by increasing the pulse duration from 100 ns to 200 ns, the thermal diffusion length also increases from 1.0 µm up to 1.4 µm (calculated with ${\rm{K}}=23\frac{W}{m\cdot K}$; $\rho =4500\frac{kg}{{m}^{3}}$ and ${{\rm{c}}}_{{\rm{p}}}=544\frac{J}{kg\cdot K}$ for Ti). Thus, for the longer pulses, a thicker layer of molten material is produced decreasing the final roughness. This effect has already been demonstrated by different authors, meaning that a larger melt pool leads to a better smoothing of the surface^37^. In addition, the observed corona around the ablated crater is formed due to material transport, which is caused at moderate and high laser fluences by the recoil pressure (also called piston mechanism)^38,39^. The piston mechanism causes lateral material transport by pressure differences at the surface of the liquid layer, leading to lateral material flow towards the region of lower pressure. The melt resolidifies at the edges and a corona-like geometry is formed around the crater. Based on the previous described results, for the rest of the experiments, the pulse duration for producing the large patterns was fixed to 200 ns.

The round shape of a crater and the homogeneous formation of the corona are mainly determined by the precise control of the positions where the laser pulses reach the surface by the used galvano scanner and the applied laser fluence^40^. The positioning accuracy can be affected by temperature changes and controlling errors of the mirrors as well as by synchronization of the scanner mirror motion with the clock of the laser source. In this work, a non-synchronized on-the-fly technique was used and no optimization regarding positioning errors was done. Thus, the position of the produced craters between two arrows could not be perfectly controlled, but the same density of features has been produced.

To quantitatively describe the quality of the produced patterns, depending on the used laser fluence, the mean structure height of the craters as well as their variation was studied. Thus, the structure height error can be used as an indicator for homogeneity of the whole structured surface. As a second parameter, the circularity of the craters was also measured. A perfect round shape indicates a well-defined corona geometry which means that the crater surface is free of defects, such as particles coming from the melt-front.

As mentioned before, higher structure might improve the adhesion of the cells to the implant surface as well as improving the mechanical damage resistance of the small DLIP features to be produced in a second step.

**Figure 2:**
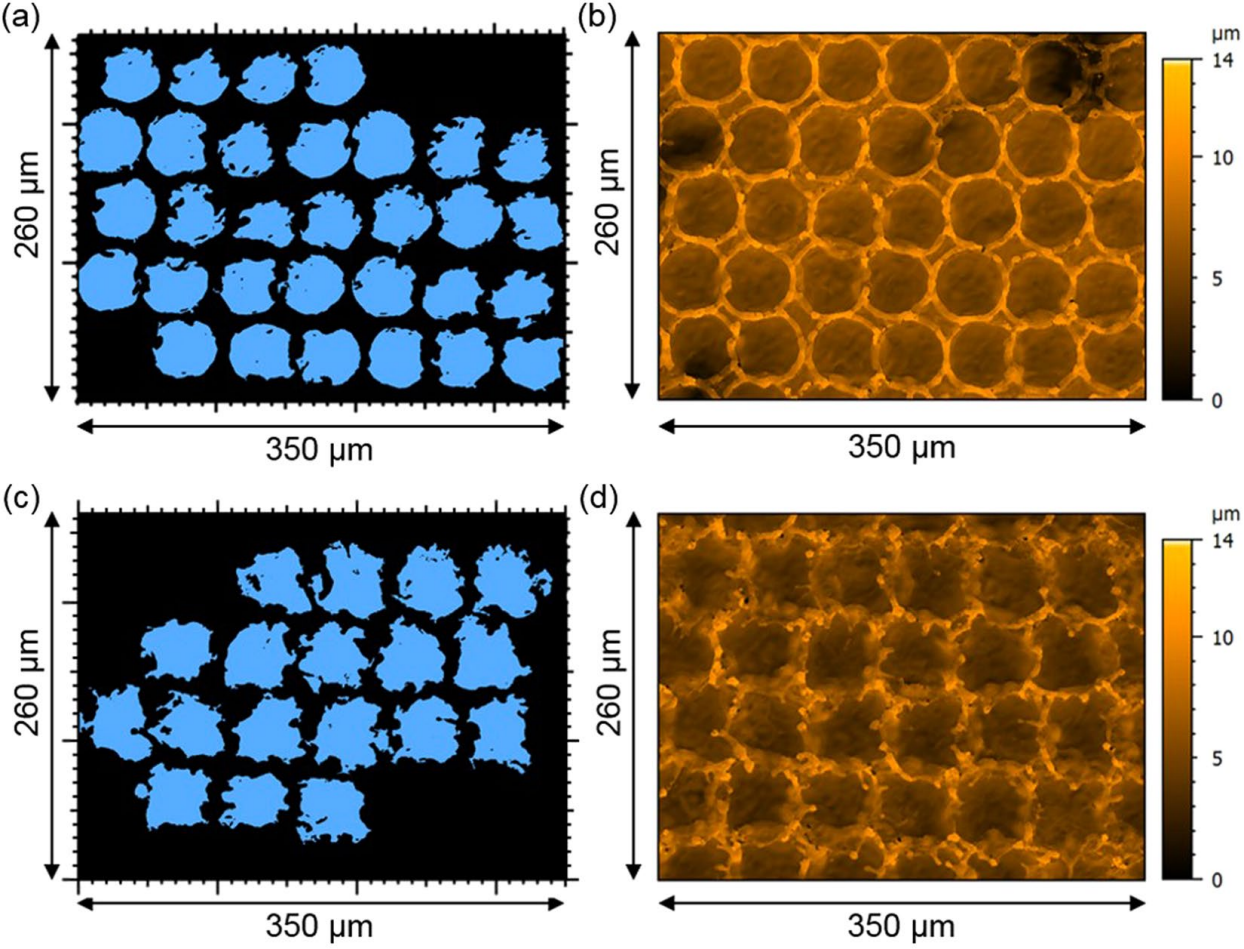
Analysis of the structure topography fabricated using DLW method with a pulse duration of 200 ns and fluences of (**a**,**b**) F = 5.9 J/cm^2^ and (**c**,**d**) F = 7.3 J/cm^2^. (**a**,**c**) Show bicolored images of the patterns where the blue color represents the area which is lower than the mean plane of the topography profile. (**b**,**d**) Show the corresponding topography image for (**a**,**c**), respectively.

In Fig. 2, bicolored and topography images of DLW structured surfaces produced using fluences of F = 5.9 J/cm^2^ (Fig. 2a,b) and F = 7.3 J/cm^2^ (Fig. 2c,d) are shown. The bicolored images represent in blue the area below the mean plane of the measured topography profile, which is associated with the formed craters. In order to analyze the produced craters from the bicolored images, a filter had to be used, consisting on a circularity criteria of 0.3 (1 means perfect circular geometry) and a minimal crater diameter criteria of 10 µm. Also, it was necessary to exclude from the analysis the craters at the edge of the image, since they were not complete.

**Figure 3:**
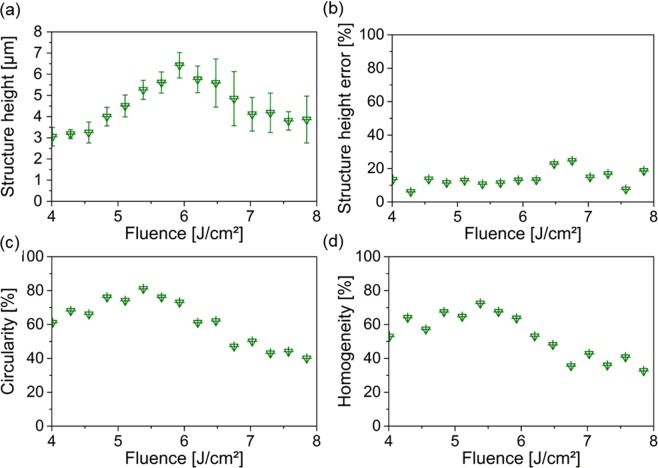
Structure analysis of DLW patterns depending on the used fluence. (**a**) Shows the structure height, (**b**) the relative structure height error, (**c**) the relative circularity and (**d**) the relative homogeneity depending on the used fluence.

Comparing Fig. 2a,c, it is possible to see that for the lower fluence of 5.9 J/cm^2^ more regular craters were formed than for the 7.3 J/cm^2^ laser fluence. The same conclusion can be obtained by analyzing the corresponding topography images (Fig. 2b,d), where craters with well-defined coronas were achieved for the lower fluence. In addition, for the higher laser fluence, several defects can be observed. A quantitative analysis of the obtained pattern quality using the parameters mentioned before as function of the laser fluence is shown in Fig. 3.

As it can be seen in Fig. 3a, the development of the structure height *h* with the laser fluence was studied for fluences higher than the ablation threshold, ranging from F = 4.0 J/cm^2^ up to F = 8.1 J/cm^2^. Between 4.0 J/cm^2^ and 5.9 J/cm^2^ the structure height of the craters increased almost linearly, ranging from h = 3.1 ± 0.4 µm up to h = 6.4 ± 0.6 µm. Using higher fluences, the structure height decreased down to h = 2.9 ± 1.1 µm, for a fluence of 8.1 J/cm^2^.

In order to quantitatively determine the structure homogeneity, the structure height error *Err%* (Fig. 3b) was calculated using Eq. 2, which expresses the ratio of structure height variation (*SD*) to the measured mean height *h*:

2 $$Err \% =100 \% \cdot \frac{SD}{h}$$

The obtained results show that in the fluence regime of the almost linearly increasing structure height, the structure height error remains almost constant between 6% and 13%. For higher fluences, higher structure height errors were measured, between 8% and 25%.

As an additional parameter for describing the quality of the crater geometry, the circularity of the craters Λ%_*circ*_ was calculated, which represents the ratio between the crater area A_*crater*_ and the area of a disk whose diameter corresponds to the greatest distance between two points of the ablated crater A_*Disk*_:

3 $${\rm{\Lambda }}{{\rm{ \% }}}_{circ}=100{\rm{ \% }}\cdot \,\frac{{A}_{crater}}{{A}_{Disk}}$$

When this value is close to 100%, it means that both areas are identical and thus corresponding to a perfect shaped circle.

Similar to the structure height development, the calculated circularities show a linear increase up to a maximum of 81% at a fluence of F = 5.4 J/cm^2^ and decreases for higher fluences.

In order to unify both parameters for describing the homogeneity of the large scaled structures, we combine the information from the structure height error and the circularity obtaining the relative homogeneity ratio *H%* according to Eq. 4:

4 $$H{\rm{ \% }}=\frac{(100{\rm{ \% }}-Err{\rm{ \% }})\,\cdot {\rm{\Lambda }}{{\rm{ \% }}}_{circ}}{100}$$

By plotting *H%* as function of laser fluence, a similar behavior as for the structure height error (Fig. 3b) and the circularity (Fig. 3c) was calculated as shown in Fig. 3d. The optimum was also achieved for a fluence of F = 5.4 J/cm^2^ with H% = 72%.

The structure height of the most homogenous pattern at F = 5.4 J/cm^2^ was 5.3 ± 0.5 µm which is 83% of maximum achieved structure height at F = 5.9 J/cm^2^.

After the laser experiments, it was also analyzed if the laser treatment affected the surface chemistry of the treated surfaces. This was qualitatively performed by EDX measurements. In the case of the untreated reference, the Ti and O content were 85 ± 1% and 12 ± 1%, respectively. In the case of the laser treated sample (at F = 5.9 J/cm^2^), similar values were measured (Ti = 88 ± 1% and O = 9 ± 1%) which means the single pulse DLW process did not affect significantly the chemistry of the titanium samples.

Although the highest homogeneity ratio was obtained for the sample treated at a fluence of 5.4 J/cm^2^, we decided to perform the rest of the DLW experiments at a fluence of 5.9 J/cm^2^, since the highest patterns could be obtained (11% higher). It has to be mentioned that homogeneity ratio of the Ti surface at 5.4 J/cm^2^ did not considerably differ from the optimal case.

### Fabrication of hole-like structures using DLIP

**Figure 4:**
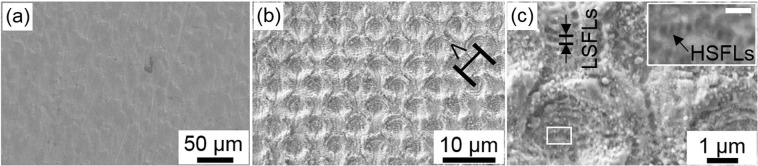
SEM images of titanium surfaces: (**a**) untreated Ti surface, (**b**) DLIP processed hole-like pattern with a spatial period Λ of 5 μm processed using a fluence of 0.93 J/cm^2^ and 5 laser pulses, (**c**) higher magnification of (**b**) with an inset to show the occurrence of Low Spatial Frequency and High Spatial Frequency Laser-Induced Periodic Surface Structures (LSFLs and HSFLs).

In order to evaluate the possibility of producing the smaller hole-like patterns using the 4-beam interference setup, the pure titanium samples were treated (without prior DLW processing). In Fig. 4, both the unstructured surface and hole-like structures produced with a 5 µm spatial period are shown. The average structure height h of the produced patterns was 1.1 ± 0.3 µm. In this case, the used fluence was 0.93 J/cm^2^ and five laser pulses were employed (pulse number N = 5). For the structuring strategy, the samples were translated horizontaly and verticaly with hatch distances H∆x and H∆y of 39.5 µm and 38.5 µm, respectively (see experimental section).

In addition to the hole-like periodic pattern (with 5 µm spatial period), also smaller features appeared on the laser treated surface. In Fig. 4c, an arrangement of periodic line patterns with a lateral spacing of approximately 400 nm on top of the interference structure are evident. Furthermore, these structures are oriented perpendicular to the polarization of the laser beam. Due to measured size, orientation and by taking into consideration that the used laser wavelength was 532 nm, these features can be identified as Laser-Induced Periodic Surface Structures (LIPSS) with a low spatial frequency (LSFLs) in agreement with Skolski *et al*.^41^. These features have been also seen in the past on other metallic surfaces treated with ps-DLIP^41,42^. In addition to the LSFLs, also another periodic array oriented parallel to the beam polarization could be observed between the LSFLs (see inset in Fig. 4c). These features are significantly smaller than the LSFL features, with a repetitive distance of approximately 150 nm. Due to the size, orientation and periodicity of these features, they can be classified as high spatial frequency LIPSS (HSFLs)^41^.

**Figure 5:**
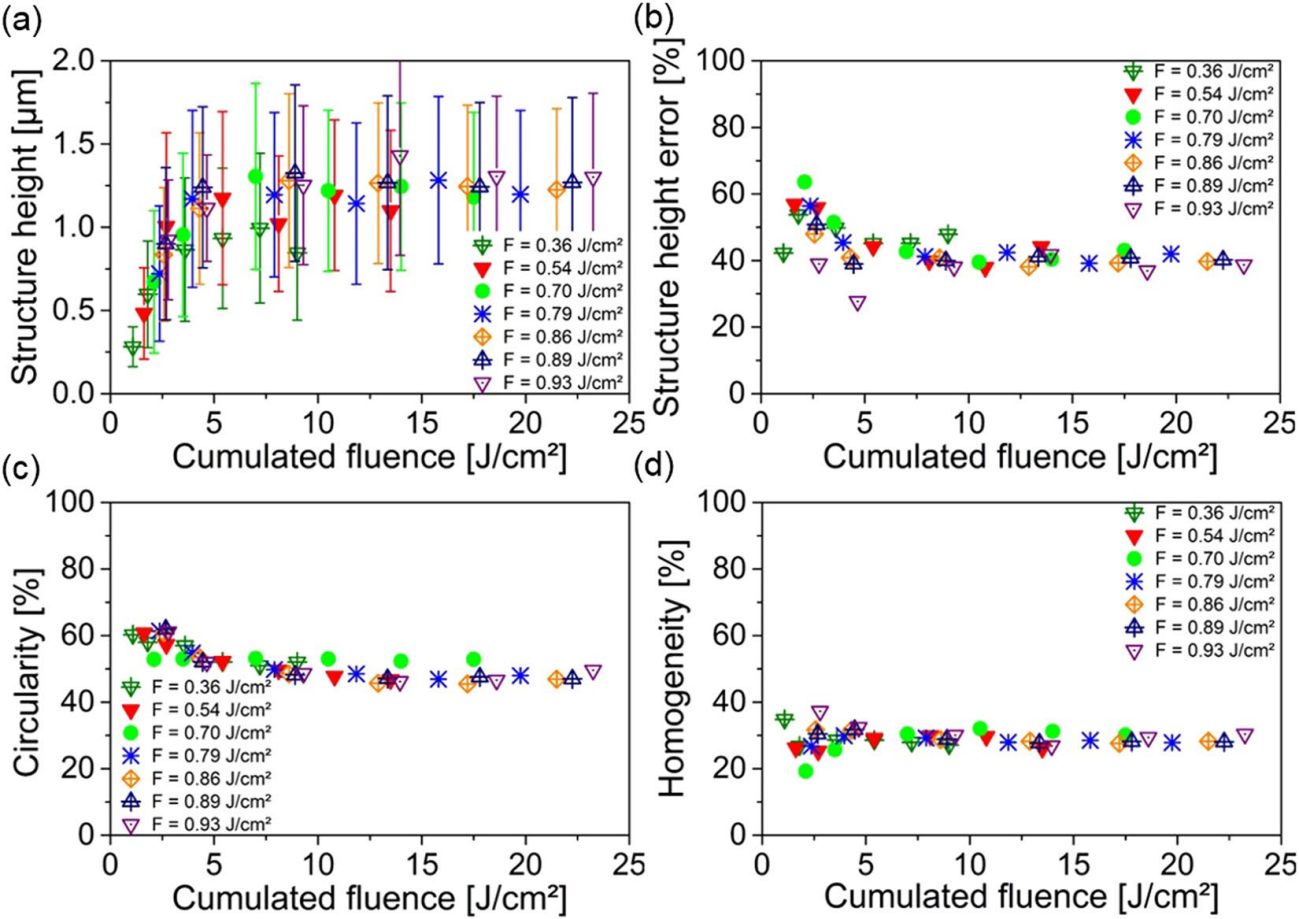
Structure analysis of single DLIP patterns depending on the used cumulated fluence. (**a**) Structure height, (**b**) Relative structure height error, (**c**) Relative circularity and (**d**) Relative homogeneity depending on the used cumulated laser fluence.

Also for the DLIP produced patterns, a quantitative analysis of the pattern quality was performed, in this case depending on the cumulated laser fluence *F*_*cum*_, as shown in Fig. 5. The cumulated fluence *F*_*cum*_ can be calculated using Eq. 5, and describes the total energy per unit of area that has been used in a certain area to treat the surface:

5 $${F}_{cum}=F\cdot N$$

where *F* is the laser fluence of each individual laser pulse and *N* is the pulse number. Regarding the structure height development and the structure height error (shown in Fig. 5a,b, respectively), a very significant deviation in the structure height (28% to 63%) for all used cumulated fluence was measured. A possible reason for this high deviation can be related to the initial surface roughness (high peak-to-valley height) of the untreated titanium surface. The average structure height, achieved using cumulated fluences ranging from 4.0 J/cm^2^ up to 23.3 J/cm^2^, varied between 0.9 µm and 1.4 µm, which was up to 5 times less than the peak-to-valley height roughness of the untreated surfaces.

On the other hand, the mean values of the structure heights obtained for the 5 µm hole-like periodic pattern increased up to 1.1 µm for F_cum_ = 4.0 J/cm^2^. By using higher laser fluences, the structure height remained constant.

The circularity of the DLIP structure was also evaluated. As it can be seen in Fig. 5c, the circularity decreases slightly with increasing cumulated fluence from 63% to 46%. Finally, using Eq. 5, the homogeneity ratio was calculated obtaining almost a non-dependency with the used processing parameters at a constant level of H% = 29 ± 3%.

### Processing of hierarchical crater and hole-like structures using DLW and DLIP

**Figure 6:**
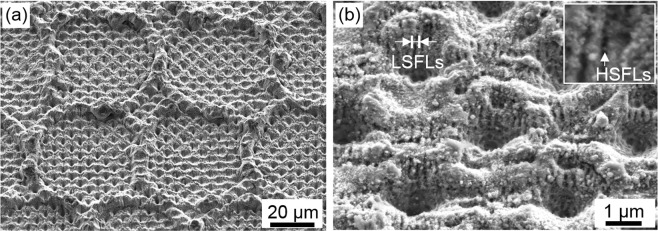
SEM images of a hierarchical surface structure of a DLW-DLIP treated Ti-surface. The DLW craters were produced using a pulse duration of 200 ns and a fluence of F = 5.9 J/cm^2^. For the DLIP features, the used cumulated fluence was F = 4.7 J/cm^2^. (**b**) High magnification image showing also the LIPSS features (LSFLs in the image and HFSLs in the inset). (titling angle: 30°).

Finally, we proceed to produce the hierarchical surface patterns by combining DLW and DLIP. In Fig. 6, representative SEM images of a hierarchical DLW and DLIP treated titanium surface are presented. The DLW crater-like structures were fabricated using a fluence of F = 5.9 J/cm^2^ and a single laser pulse per crater. After that, the DLIP structures were produced, using a fluence of F = 0.93 J/cm^2^ and a pulse number N of 5.

The hatch distances were H∆x = 39.5 µm and H∆y = 38.5 µm (see experimental section for more information). The resulting hole-like DLIP pattern had a spatial period of 5 µm and a structure height of h = 0.9 ± 0.1 µm, which was measured into the DLW craters. The topography of the Ti samples of Fig. 6a, show that the DLIP pattern is visible into the craters as well as on their corona and between them. It can be also seen, that the DLW pattern was not destroyed or deteriorated by the DLIP treatment. In Fig. 6b, the obtained topography is shown at a high magnification. Similarly like in the previous case, LSFLs and HSFLs are present on the ps-DLIP treated surface and have the same spatial periods and orientation like those fabricated by the single DLIP process (~400 nm and 150 nm for LSFL and HSFL, respectively). The images also show a better definition of the DLIP structures, compared to the structures shown in Fig. 4.

A quantitative analysis of the DLIP pattern quality in the DLW craters depending on the cumulated laser fluence *F*_*cum*_ was also performed in this case. Similarly like in the previous cases, the same criteria were used.

**Figure 7:**
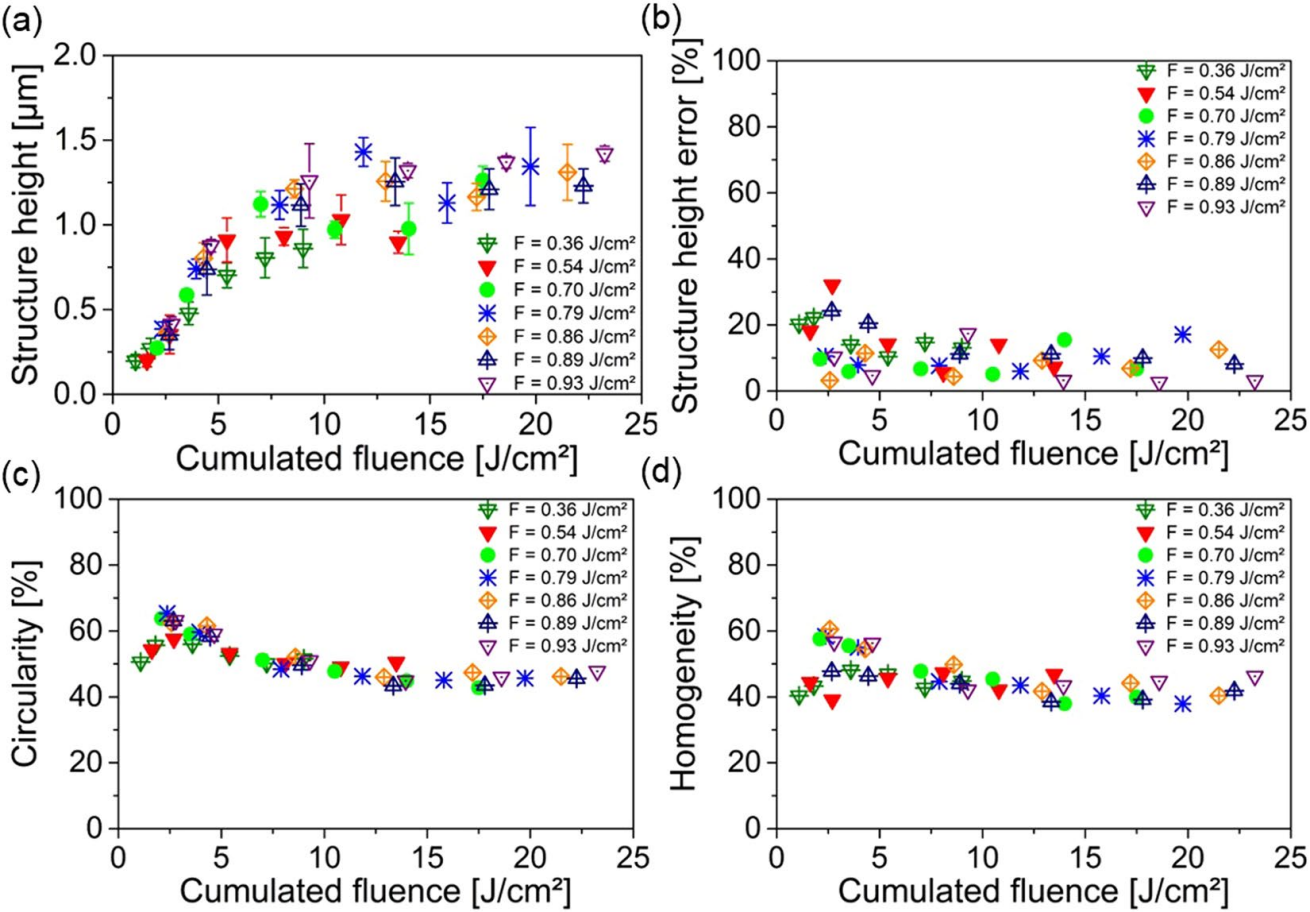
Topography analysis of DLIP patterns produced into the DLW craters depending on the used cumulated fluence. (**a**) Structure height, (**b**) relative structure height error, (**c**) relative circularity and (**d**) the relative homogeneity depending on the cumulated fluence.

Figure 7a shows the average structure height as function of the cumulated laser fluence. It can be seen, that the structure height increases from 0.2 ± 0.1 µm up to 1.4 ± 0.1 µm for cumulated fluences F_cum_ ranging from 1.1 J/cm^2^ up to 11.9 J/cm^2^. By using higher fluences, no increase in structure height was observed. This behavior was very similar to the results reported in the previous section.

Regarding the structure height error (see Fig. 7b), it can be seen that the deviation in the structure height at each cumulated fluence was significantly smaller compared to the single DLIP process. In average it was reduced from 44% for single DLIP structures down to 11% for the DLIP structures in the DLW craters. The reason for the lower standard deviation of the measured heights can be related to the reduced peak-to-valley height roughness into the large size craters produced by the DLW treatment. Similar results have been reported by Alamri *et al*., where a significant improvement of the pattern morphology was observed for hole-like structures produced using three-beam DLIP on laser-flattened steel surfaces. However, further investigations are required in the future for a quantitative analysis of the influence of the initial surface roughness with the structure pattern quality.

The results regarding the circularity of the produced DLIP patterns are shown in Fig. 7c. The results show that the optimal circularity was achieved using relative low cumulated fluences between 2.5 J/cm^2^ and 4.7 J/cm^2^. In this regime, up to 65% of circularity could be reached (for F = 0.79 J/cm^2^ and N = 3). Using higher cumulated fluences, the circularity decreased down to 43% (F = 0.70 J/cm^2^ and N = 25 pulses). Explanations for this behavior could be imperfections in the DLIP interference area or lacking overlap congruence of the interference areas. Finally, an optimal homogeneity ratio could be determined for cumulated fluence between 2.5 J/cm^2^ and 4.7 J/cm^2^, with a highest homogeneity of H% = 61% for F_cum_ = 2.6 J/cm^2^ (F = 0.86 J/cm^2^ and N = 3 pulses). The average homogeneity over all structures was 46 ± 6%, which is 1.6 times better compared to the single DLIP process.

Also in this case, EDX measurements were performed on the treated surfaces for determining the influence of the laser treatment on the surface chemistry. For a Ti sample treated with a cumulated fluence F_cum_ of 4.7 J/cm^2^, an increase in the oxygen content in the reactive layer was observed compared to the untreated reference and the DLW processed surfaces. For the mentioned fluence, an oxygen content of 28 ± 1% was measured (66 ± 2% for Ti). The difference between both laser processes can be explained as follows. Due to the small thermal diffusion length in Ti for the 70 ps pulses (25 nm, calculated using Eq. 1), the very concentrated heat at the surface can ablate and vaporize the metallic material at the interference maxima positions. The hot metal vapor can later oxidize in contact with the atmospheric oxygen and is pushed towards the interference minima regions due to the pressure vapor difference. Then, the material is partially deposited at the minima regions. The observed titanium oxidization could be promising in terms of long term stability of the dental implants, as reported by McQueen *et al*. and Sundgren *et al*.^12,13^.

### Wear and bacterial adhesion tests on hierarchical surface patterns

Wear test were performed on the processed Ti surfaces with DLW and DLIP methods, in order to proof the capability of the large pattern to protect the hole-like structure as well as the titanium oxide layer inside the craters from mechanical damage. These tests were performed using a ball-on-disk tribometer (see experimental section). For comparison objectives, also a DLIP treated Ti samples was evaluated exactly at the same conditions (without DLW treatment).

**Figure 8:**
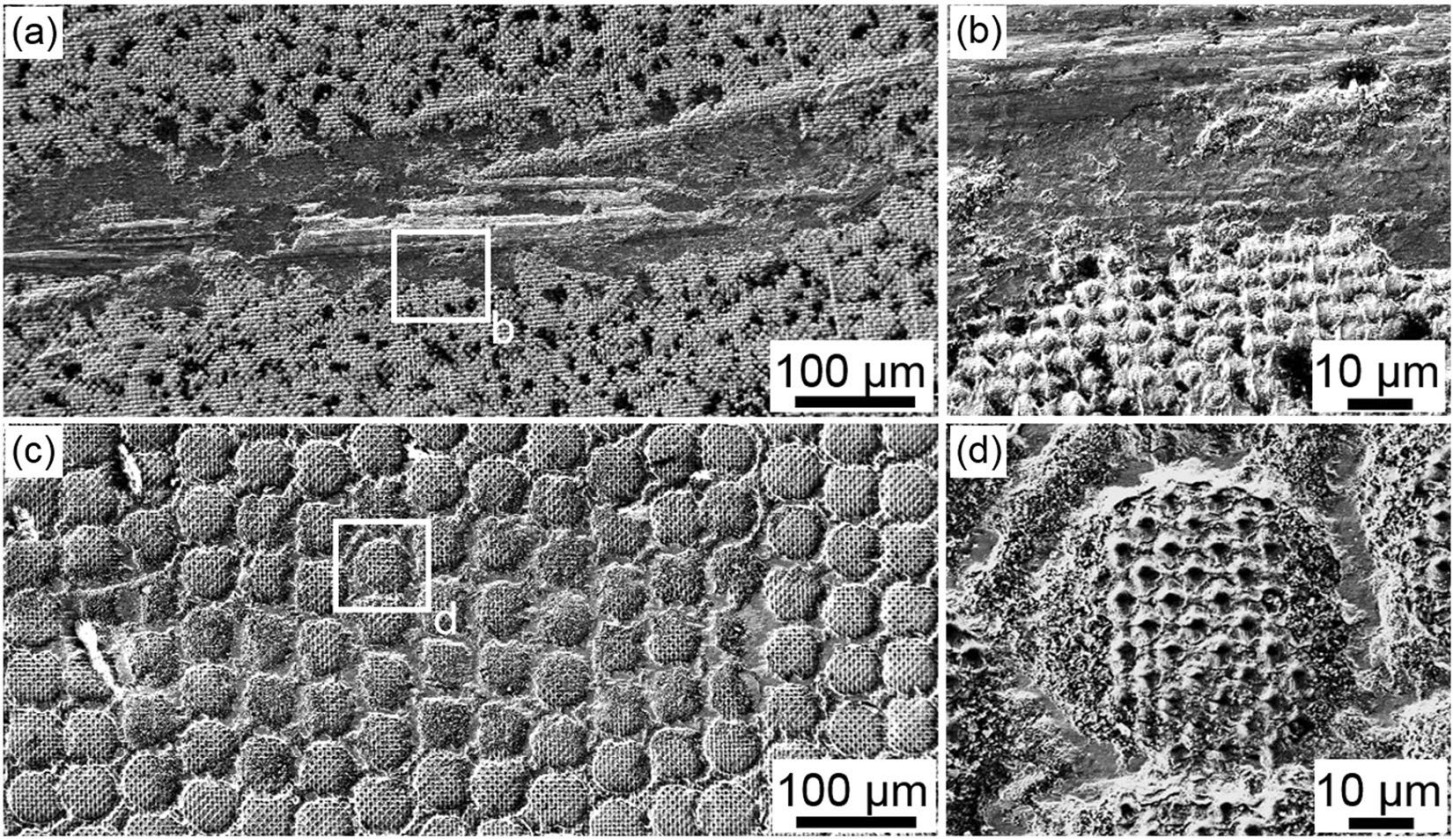
SEM images of wear tracks achieved by a ball-on-disk test. The used 100Cr6 ball had a diameter of 6 mm and the normal force was kept constant at 0.8 N. (**a**,**b**) Wear track on DLIP structured surface; (**c**,**d**) Wear track on hierarchical DLW and DLIP structured Ti sample.

SEM images of the resulting wear tracks are shown in Fig. 8. The DLIP patterns were fabricated using a fluence F of 0.93 J/cm^2^ and a pulse number N of 5 in both cases. The resulting structure height of the DLIP produced craters were h = 1.1 ± 0.3 µm for single DLIP and h = 0.9 ± 0.1 µm for DLIP in DLW craters.

In the case of the sample treated only with DLIP (Fig. 8a,b), the periodic 5 µm hole-like geometry was totally destroyed at the sliding area in contact with the ball. The average wear track depth was for this case 1.4 ± 0.4 µm. On the contrary, the DLIP structures produced into the DLW craters were not affected by the wear test (see Fig. 8c,d). In this case, the ball only affected the coronas of the craters produced by DLW, which denotes the absence of mechanical contact between the ball and the DLIP pattern. The average structure height of the crater coronas was reduced from 6.4 ± 0.6 µm to 5.1 ± 0.9 µm due to the abrasive wear.

Finally, bacterial adhesion tests were performed on the untreated and laser treated titanium samples. Since the main objective was to prove the multifunctional character of the DLW-DLIP treated samples, only the performance of these substrates were tested. The used laser fluence F and pulse number N for the DLIP process were 0.20 J/cm^2^ and 10, respectively, obtaining a cumulated fluence F_cum_ of 2.0 J/cm^2^. For this condition, a structure height of h = 0.4 ± 0.1 µm was achieved. The substrates were incubated with *E*. *coli* cells (rod-shaped with about 1 µm width and 2–3 µm minimal length) in growth medium for 24 h. Subsequently, the relative area covered by adherent cells was quantified (see experimental section).

**Figure 9:**
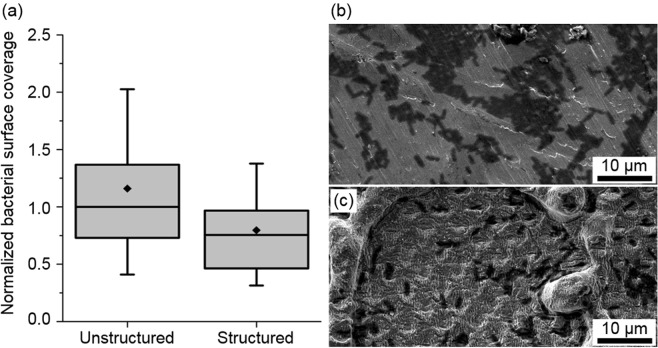
Bacterial adhesion test on titanium surfaces. (**a**) shows the normalized bacterial surface coverage for *E. Coli* cells after 24 h. The box–whisker plots present half of the data points within the box and 100% within the whiskers. The black continuous lines and black diamonds within the boxes mark the median and mean, respectively. The values are normalized to the median colonized surface of the unstructured area of the samples. (**b**) shows a SEM image of bacteria colonization (dark areas) on the unstructured surfaces and (**c**) on the structured surface with reduced bacterial colonization.

Figure 9 shows the measurement of the normalized bacterial surface coverage as well as SEM images of the untreated and the structured surfaces with adhered bacteria.

In the box-whisker plots in Fig. 9a, the surface covered by bacteria was normalized to the median colonized surface of the unstructured area. The average bacterial adhesion on the DLW-DLIP combined pattern was reduced by about 30%. Regarding the SEM images (Fig. 9b,c), a reduced bacterial colonization (dark areas) can be seen on the structured surface compared to the unstructured reference. In the past, it has been reported that both roughness and hydrophobicity of a surface can significantly affect bacterial colonization. Furthermore, it is difficult to distinguish which effect dominates when surfaces of different materials are compared in terms of their colonization properties^22,23,43,44^. Concerning topographical aspects, the obtained LSFL exhibit a spatial period of approximately 400 nm (see Figs 4c or 6d) what can explain the lower adhesion of bacteria, since their feature size is significantly smaller than the bacterial cell dimension (~800–2000 nm). This result is in agreement with previous recent research works, where a reduced bacteria retention was observed on LIPSS with spatial periods between 0.5–0.9 µm^22,23^. Concerning the crater-like geometry produced by DLW (~50 µm diameter), we believe that they might not reduce bacterial retention, as others have even observed a slight increase in bacteria adhesion on large-scale structures^23,45^. Differently, the 5 μm hole-like structure generated by DLIP could also contribute to bacterial-repellency, as Helbig *et al*. have observed for *E. Coli* bacteria on such structures made on photoresist surfaces^21^. Concerning surface wetting, Lutey *et al*. discussed an enhanced bacteria-repellency of surfaces that show already less bacteria adhesion^23,45^. In this study, however, the surfaces were hydrophilic (water contact angle of 32 ± 8°) and therefore the wetting of the surface should not strongly affect the bacterial-repellency.

## Conclusions

Titanium grade 4 substrates were treated using DLW and DLIP processes to produce hierarchical multifunctional surfaces. In the case of DLW, variation of the laser fluence from 2.0 J/cm^2^ to 8.1 J/cm^2^ permitted to produce crater-like patterns with a diameter of approximately 50 µm and heights from 1.7 ± 0.6 µm to 6.4 ± 0.6 µm. In addition, using pulses with a duration of 200 ns, it was possible to reduce the initial peak-to-valley roughness from 5.2 µm to 2.6 µm.

Also, a criterion to evaluate the homogeneity of the produced patterns consisting on the measurement of the structure height variation and circularity was developed. It was found, that the most homogenous patterns could be produced at a laser fluence of 5.4 J/cm^2^.

Using the DLIP method with a 4-beam interference optics, 5 µm spatial period hole-like patterns were produced on both the non-treated and DLW treated Ti substrates. By varying the laser fluence and the pulse number, the pattern homogeneity and structure height were controlled. Due to ultrashort pulse treatment for the DLIP process (70 ps), also LIPSS features (HSFLs and LSFLs) were produced with spatial periods around 400 nm (HSFLs) and 150 nm (LSFLs). In the case of the Ti surfaces treated first with DLW and later with DLIP, a higher quality of the DLIP features was observed.

EDX measurements of the laser processed samples revealed a significant increase of the oxygen content by the ps-DLIP treatment only.

The hierarchical textured Ti surfaces showed both a reduced wear as well as a lower bacterial adhesion (~30%) when using *E*. *Coli* bacteria. In consequence, we could demonstrate the advantage of equipping surfaces with features of different length scales in order to obtain multifunctional surfaces.

## Experimental

### Samples preparation

Titanium grade 4 samples with a diameter of 16 mm and a thickness of 2 mm were used. The samples were cut from a rod by wire-cut EDM and were grinded afterwards with maximum 1200 grit emery paper. Before and after the laser treatments, all substrates were cleaned in ultra-sonic bath with pure ethyl alcohol (C2H5OH) for 5 minutes. The samples for bacterial adhesion test were shrink-wrapped for transport with bacteria proof and heat sealable polyester-polypropylene foil MELAfol (EN 868-5) from MELAG Medizintechnik.

### DLW setup

**Figure 10:**
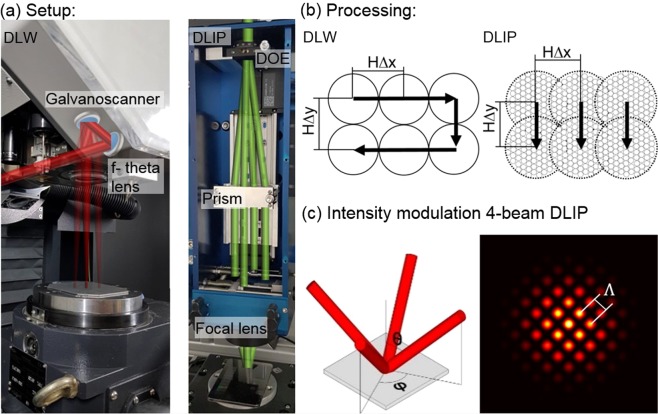
DLW and DLIP configurations. (**a**) Used DLW and DLIP systems. The surfaces were functionalized using the process strategies presented in (**b**). (**c**) Resulting intensity modulation ranging from black (no intensity) to yellow (high intensity) for the overlap of 4 beams.

A pulsed nanosecond ytterbium fiber laser (YLPN, IPG Photonics) with a wavelength of 1064 nm, adjustable pulse waveforms in the range of 4–200 ns and a pulse repetition rate ranging from 2–1000 kHz was used. The laser beam is guided meandering with a laser scanner (Scanlab GmbH) with a focal length of 254 mm over the substrate, which leads to a beam waist ${\omega }_{0}$ of 42 µm (Fig. 10a). In this case, the samples were irradiated with pulse durations *τ* of 100 and 200 ns and laser fluences *F* from 2.0 J/cm^2^ to 8.1 J/cm^2^. The hatch distances in x and y direction (H∆x and H∆y) were equal (50 µm) to the diameter of the ablated craters (see Fig. 10b).

### Four-beam DLIP setup

The periodic patterns were generated by interfering 4 laser beams emitted by a pulsed Nd:YAG laser system (neoMOS 70 ps, neoLASE GmbH) whose 1064 nm fundamental wavelength is frequency doubled to 532 nm. The pulse duration was 70 ps. Figure 10a shows the utilized interference optics (Fraunhofer IWS, Dresden, Germany). The incident beam is divided by a diffractive optical element (DOE) into four coherent beams of comparable intensities which are subsequently collimated by a four side prism. Finally, the beams were overlapped on the substrate surface using an aspherical lens with a focal length of 60 mm. The interference of the four laser beams causes a dot-like intensity modulation, which spatial period can be calculated by Eq. 6 and its distribution is shown in Fig. 10c. The angle of incidence *θ* defines the spatial period Λ between two intensity maxima. The azimuthal angle *φ* in the spherical coordinate system is 90° between two adjacent beams.

6 $${\rm{\Lambda }}=\frac{\lambda }{\sqrt{2}\,\cdot \,sin\,\theta }$$

By setting the incident angle to θ = 4.31°, a spatial period Λ of 5.0 μm was obtained. The diameter of the interference region was ~70 µm. The hatch distances in x and y direction were set to 50% of the diameter of the interference region to obtain a more homogeneous pattern. The structure depth and morphology were controlled by the laser fluence and the number of pulses, which were varied from 0.36 J/cm^2^ to 0.93 J/cm^2^ and from 3 to 25 pulses. This resulted in cumulated fluences *F*_*cum*_ between 1.1 J/cm^2^ and 23.3 J/cm^2^.The laser frequency was set to 1 kHz for studying the structure development and increased to 50 kHz for the production of samples used in bacterial adhesion test.

### Surface characterization

The surface morphology of the DLW and DLIP processed samples was analyzed using both, a scanning electron microscope (ZEISS Supra 40VP) and a confocal microscope (Sensofar S Neox). In the case of the confocal microscopy analyses, a 50x magnification objective was used, having a lateral and a vertical resolution of 170 nm and 3 nm, respectively. For the quantitative analyses of the homogeneity parameters, the software Sensomap 7.3 (Sensofar) was used. The chemical composition of the non-treated and laser treated Ti-surfaces was analyzed using energy dispersive X-ray spectroscopy (EDX) at 5 keV excitation energy (Quantax, Bruker).

### Wear test

The wear experiments were performed using a ball-on-disc tribometer (CSM Instruments, Nanotribometer) in linear sliding mode. The normal load was kept constant at 0.8 N and a 6 mm ball (of steel 100Cr6) was used. The sliding velocity was set to 5 mm/s. The length of the sliding area was 1 mm and 300 cycles were used. During the tribological tests, the temperature was 22 °C, with a relative humidity of 30%. Before each experiment, the ball and sample were cleaned with pure ethyl alcohol (C_2_H_5_OH).

### Bacterial adhesion test

*Escherichia coli* (strain W 3310) were grown overnight in Luria- Bertani (LB) medium. The overnight culture was adjusted to an optical density of 0.001 (OD_600_) and incubated with the samples at 37 °C under gentle shaking (80 rpm). After 24 h the samples were fixed in paraformaldehyde, washed with PBS and MilliQ, and dried with nitrogen gas. After sputtering with a 10 nm gold layer (BALZERS SCD 050 Sputter Coater) the unstructured and hierarchically structured part of the titanium surface were imaged with SEM (FEI ESEM XL30 FEG). The experiments were performed three times with three samples respectively. Four images per structured and unstructured part of each sample were evaluated with ImageJ (v 1.52a Wayne Rasband, NIH USA) by measuring the area covered with bacteria. The results were normalized to the median of the coverage on the unstructured sample parts.

## Data Availability

The datasets generated during and/or analyzed during the current study are available from the corresponding author on reasonable request.
